# Generation of Novel Fuels Optimized for High-Knock Resistance with a Long Short-Term Memory Model

**DOI:** 10.1021/acs.energyfuels.5c01155

**Published:** 2025-06-30

**Authors:** Sergey Anufriev, Paul Hellier, Nicos Ladommatos

**Affiliations:** Department of Mechanical Engineering, 4919 University College London, Roberts Building, Torrington Place, London WC1E 7JE, U.K.

## Abstract

The chemical structure of fuels significantly influences the properties of ignition and energy release during combustion, making the exploration of molecular structure–property relationships a key focus for the research and development of new sustainable fuels. Given the vast combinatorial possibilities of potential fuel candidates, prioritization is essential. This study explored the use of generative modeling to propose novel molecular structures for future fuels. Specifically, the long short-term memory (LSTM) autoregressive model was fine-tuned using a hill-climb optimization algorithm to generate structures optimized for high-knock resistance. The generated compounds, unseen during training, were evaluated for their physical properties and research octane number (RON). The generated molecules contained features commonly associated with knock resistance, such as branching and aromaticity, while also uncovering unconventional structures, including oxygenates with ether linkages. This work underscores the promise of generative modeling in fuel design and highlights the strategic advantage of initiating molecular generation from predefined fragments related to known feedstocks and production processes to enhance practicality in synthesis and resource utilization.

## Graphical abstract

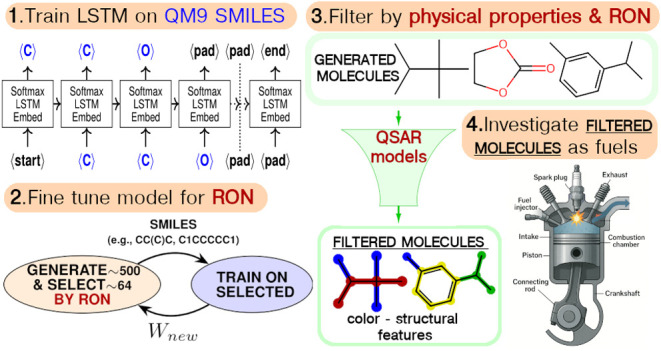

## Introduction

Global energy demand was projected to increase in the next decade,^1^ while climate change was estimated to result in economic losses of up to 10% of global GDP at +3 °C, with the most severe impacts occurring in poorer, low-latitude countries.^2^ Consequently, renewable fuels have emerged as a sustainable alternative energy source,^3^ including biofuels produced from various biomaterials such as algae, corn oil, and sugar cane and synthetic fuels such as those derived from renewable hydrogen and captured carbon dioxide.^4^ Research by^5^ demonstrated the relationship between the molecular structure of biofuels and their ignition properties.

The relationship between molecular structure and properties was widely studied in the pharmaceutical industry, where one key machine learning method used was de novo molecular design.^6^ De novo molecular design refers to the process of automatically proposing novel chemical structures that optimally satisfy a desired molecular profile.^7^ Most of the approaches used machine learning models to generate graph-based chemical structures,^8–10^ SMILES strings,^11,12^ or a combination of both.^13^ This extensive research also led to benchmarking and comparing these methods by Brown et al.^14^ and Nigam et al.^15^

In the fuel domain, de novo molecular design has been adopted^16^ to propose novel fuel molecular structures optimized for combustion properties such as the research octane number (RON).^17^ For example, a graph-based generative model in Rittig et al.^18^ used RON as the target property, generating both known high-knock-resistant compounds and a previously unknown compound that was experimentally validated. Furthermore, recognizing that real fuel compositions consist of multiple chemical structures, Kuzhagaliyeva et al.^19^ proposed designing fuel mixtures using generative machine learning. More recently, an evolutionary algorithm was introduced by Fleitmann et al.^20^ to generate fuel molecular structures by identifying optimal combinations of predefined molecular fragments that enhance knock resistance while satisfying physicochemical and combustion property constraints.

This study proposes a SMILES-based de novo design approach for future fuels using a long short-term memory (LSTM) model,^21^ fine-tuned with a hill-climb algorithm^22^ to generate high-knock-resistant compounds. The novelty lies in the application of a language model specifically tailored to generate SMILES representations for high-knock-resistant fuels. Unlike previous machine-learning-based generative studies, this approach leverages an autoregressive modeling framework, enabling molecular structure generation by sequentially appending new SMILES symbols to a fixed fragment without model retraining. In contrast, one-shot generative models such as GANs^23^ and VAEs^24^ lack this capability, making them less suitable for controlled molecular design in this context.

Oxygen was selected as the base fragment, as bioderived chemicals often contain oxygen prior to upgrading. Therefore, the unique capability of autoregressive models was utilized to generate molecules targeted by RON values plausibly produced from biomass. Furthermore, only newly generated SMILES representations (since the model is sequence-based) that were not present in the training data were evaluated for their physical properties, aligning with the goal of de novo design to create novel structures. This is in contrast to screening methods,^25^ where potential fuel candidates are limited to the SMILES data set used.

## Methodology

### Overview

In this study, molecules were represented by SMILES strings, a linear notation derived from representations of molecular structures based on graphs.^26^ Quantitative structure–activity relationship (QSAR) models were developed to predict fuel properties—including research octane number (RON), density, boiling point, viscosity, and enthalpy of combustion based on their SMILES representations.

Thereafter, a generative autoregressive LSTM model was trained on the QM9 ^27^ data set, which consists of small, stable organic molecules, to capture the syntactic and contextual patterns of SMILES representations for small organic compounds. Spark ignition fuels typically do not contain more than 9 heavy atoms, aligning with the composition of the QM9 ^27^ data set. The model was subsequently fine-tuned using a hill-climbing algorithm, which iteratively refined the model by leveraging the top-performing molecules to enhance the RON of the generated compounds.

While other fuel properties are also important in determining the performance of spark ignition engines, for example, the enthalpy of vaporization, enthalpy of combustion, viscosity, and flame propagation characteristics, RON was selected for the development of this model and as the target fuel property for novel molecule generation because of the availability of a relatively large experimentally determined data set.

The performance of these molecules was evaluated by using the developed RON QSAR model. To ensure that the generated molecules exhibited sensible physical properties, additional QSAR models for the density, boiling point, viscosity, and enthalpy of combustion were used to filter the generated compounds.

The two types of molecular generation performed in this study were initiated either from scratch or by leveraging the LSTM cell memory initialized with an oxygen atom.

### Data Sets

The research octane number (RON) data set for single-component hydrocarbons and oxygenates was compiled from the Supporting Information provided in published studies, including Whitmore et al.,^28^ vom Lehn et al.,^29^ and Abdul Jameel et al.^30^ Additionally, the data set used by Liu et al.^31^—who modeled RON—was obtained directly from the authors upon request. The research octane number data set used in this study is included in the (RON_data set.xlsx) file available in the manuscript Supporting Information. Viscosity data were sourced from a study on biofuel viscosity prediction.^32^ Data on boiling point, enthalpy of combustion, and density were retrieved from the Handbook of Thermodynamic and Physical Properties of Chemical Compounds.^33^

### Modeling Fuel Properties

The descriptor-calculation software Mordred,^34^ in combination with RDKit,^35^ was used to calculate 894 molecular descriptors from the SMILES representations of each molecule in the fuel property data sets (Table 1). To address the high dimensionality of the resulting data set, descriptors with a normalized variance below 0.001 were removed. Furthermore, highly correlated descriptors (with a coefficient of determination exceeding 95%) were eliminated, according to standard QSAR data preprocessing practices.^36^

**Table 1:** Data Set Sizes

fuel property	size
RON	362
boiling point	5549
enthalpy of combustion	2057
density	3930
viscosity	1554

Subsequently, QSAR models for fuel properties were developed using a factorial experimental design, involving four machine learning algorithms and four variable selection methods to identify the optimal combination of descriptors, algorithms, and hyperparameters. The machine learning algorithms used were multilayer perceptron (MLP), support vector machine (SVM), gradient boosting machine (GBM), and random forest (RF). Table 2 summarizes the algorithm parameters and their respective tuning ranges. The Supporting Information includes the parameters found in Table S1.

**Table 2:** Model Parameter Grid

algorithm	parameter	parameter	parameter
SVM	10^–3^ < *C* < 10^3^	10^–3^ < γ < 10^–1^	2 < *d* < 10
RF	2 < maxdepth < 64	4 < maxleafnodes < 20	5 < maxfeatures < 30
MLP	10^–5^ < lr < 10^–2^	10^–4^ < α < 10^–1^	3 < batch size < 10
GBM	2 < maxdepth < 10	5 × 10^–2^ < lr < 10^–1^	5 < maxfeatures < 30
GBM	20 < *n* trees < 300	0.2 < subsample < 0.9	

The variable selection methods included elastic net, sequential feature selector using two learning algorithms (a linear model and SVM), and an approach without variable selection.

Each data set was divided into a test set (20%), which was not used in any form of modeling. The remaining 80% of the data was used for a 5-fold cross-validation to compare the performance of the variable selection methods with the trained machine learning algorithms. Negative mean square error (NMSE) was used to measure the quality of the cross-validation. The Optuna Python module^37^ was employed to optimize the machine learning hyperparameters, maximizing the NMSE on the cross-validation.

### Generative Model

A generative model was developed using an autoregressive LSTM architecture to predict the next SMILES symbol based on the sequence of preceding symbols, enabling the generation of valid organic compounds. The model was trained on the QM9 ^38^ data set, with SMILES sequences representing molecules as inputs. Each sequence consisted of atom and bond symbols, starting with a “START” token to mark the beginning of the sequence. To ensure consistency in sequence lengths, shorter sequences were padded with “PAD” tokens. The ground truth labels were created by shifting the input sequence one step to the left, excluding the “START” token, and appending an “END” token to signify termination.

Each SMILES symbol, along with the “START”, “PAD”, and “END” tokens, was represented by a unique integer. This integer corresponded to a specific row in an embedding matrix (Figure 1), allowing the model to learn vector representations for each symbol during training.

**Figure 1:**
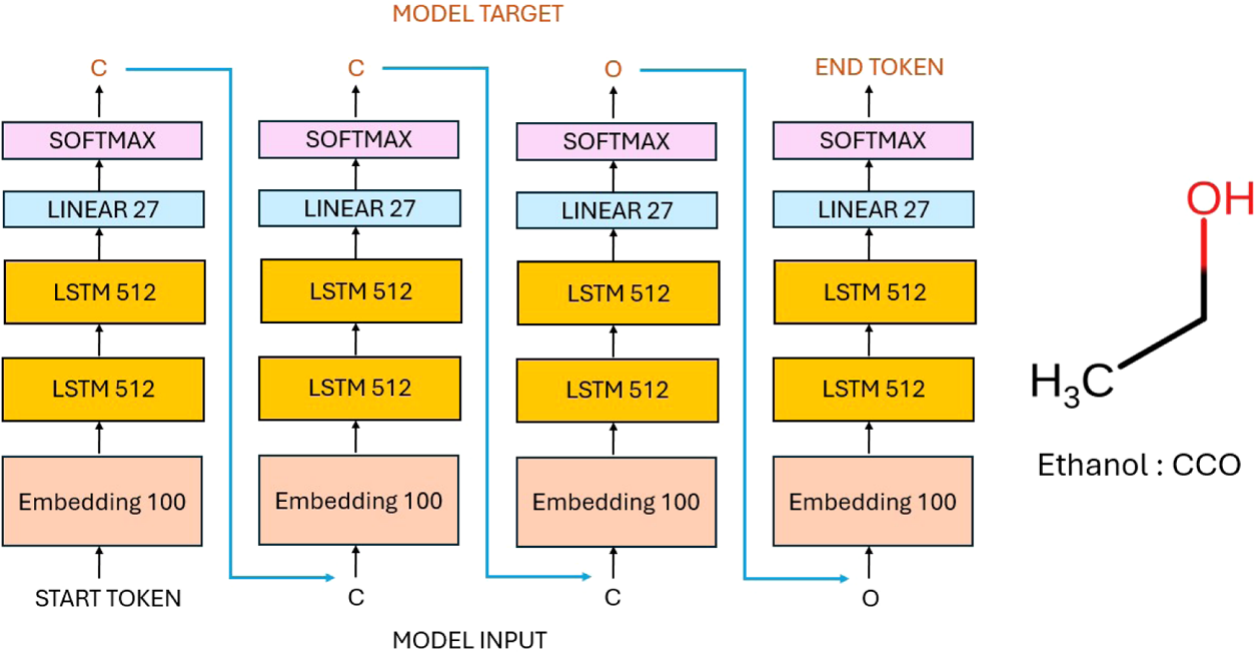
Example of an autoregressive model processing ethanol for training.

This setup enabled the model to capture the sequential dependences and contextual patterns inherent in molecular representations. For example, the SMILES sequence for ethanol was used as the input, while the corresponding output sequence was a one-symbol-shifted version of the input (Figure 1). By treating molecules as sequences, the model leveraged techniques from natural language processing to generate syntactically and semantically valid SMILES representations.

#### Model Architecture

As depicted in Figure 1, the model architecture consisted of four key components: an embedding layer, an LSTM layer with two hidden layers, a linear layer, and a softmax activation layer:

1. **Embedding layer**: this layer converts input tokens into dense vectors. It takes an input size of 27, corresponding to the number of unique symbols in the QM9 ^38^ SMILES data set plus the special tokens (start, end, and padding), and maps each token to a dense vector of size 100. This transforms the input symbols into a continuous vector space. The weights are initialized randomly, with values sampled from a uniform distribution between −1 and 1, divided by the square root of the embedding dimension.
2. **LSTM layer**: the model’s core consists of an LSTM cell with two layers. Each LSTM layer has 512 hidden units. This component processes the sequential data, capturing temporal dependencies and patterns. The LSTM layer’s weights are initialized using orthogonal initialization for weight matrices and uniform initialization for bias vectors. The hidden and cell states are initialized to zeros.
3. **Linear layer**: following the LSTM layers, a fully connected (linear) layer is used to map the LSTM outputs to the desired output size. This layer has 27 output units, corresponding to the number of unique symbols in the QM9 ^38^ SMILES data set plus the special tokens (start, end, and padding). The weights are initialized with values sampled from a uniform distribution. The range is determined by the square root of the inverse of the input size of the weight matrix.
4. **Softmax activation layer**: finally, a softmax activation function is applied to the output of the linear layer. This layer converts the raw output scores into probabilities, enabling the model to predict the likelihood of each possible symbol.

#### Training Procedure

The training was conducted over 10 epochs with a batch of 64 molecules each sampled from the QM9 ^38^ data set. Adam optimizer^39^ was used with a constant learning rate of 10^–3^, using default settings for other parameters as provided by PyTorch.^40^ A custom cross-entropy loss function was used, which computed the loss for each symbol in the sequence, excluding padding symbols by using a mask. The loss for each sequence was averaged by the number of unpadded symbols, and the average loss across the batch is returned.

#### Fine-Tuning Generative Model

To bias molecular generation toward compounds with potentially high RON values, a hill-climbing algorithm^22^ was employed to fine-tune the generative model. This algorithm iteratively adjusts the model’s weights by retraining it on a subset of generated molecules with the highest predicted RON values.

An additional constraint was introduced because the generative model was initially trained on compounds containing atoms not typically found in biofuels. If a generated compound contained such atoms, like nitrogen or fluorine, which are atypical for fuels, then the RON prediction was multiplied by −1. Similarly, invalid molecules were assigned a score of −1000. Both nonfuel-like and invalid molecules were thus excluded from selection as top-performing candidates. The exact implementation of the hill-climb algorithm is provided in Algorithm 1.
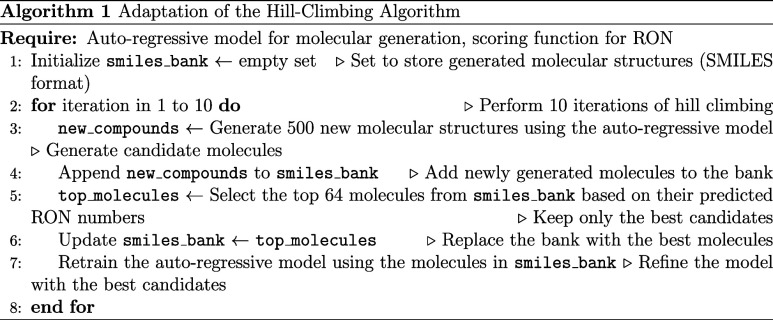


### Molecular Generation

The molecules were generated recursively using eqs 1–4, where each step *t* produced a symbol *s*_*t*_ from the SMILES vocabulary, including “PAD” and “END” tokens

1 $$h_{t},\;c_{t}=\mathrm{LSTM}\left(E\left[s_{t-1}\right],\;h_{t-1},\;c_{t-1}\right)$$

2 $$y_{t}=W_{\mathrm{out}}\cdot h_{t}+b_{\mathrm{out}}$$

3 $$P\left(s_{t}=k|s_{1},\;...,\;s_{t-1}\right)=\mathrm{softmax}\left(\frac{y_{t}}{T}\right)$$

4 $$s_{t}=\mathrm{sample}\;\left(P\left(s_{t}|s_{1},\;...,\;s_{t-1}\right)\right)$$

Here, *E*[*s*_*t*–1_] represents the embedding of symbol *s*_*t*–1_, and **h**_*t*_ and **c**_*t*_ are the LSTM states. The output **y**_*t*_ is computed via a dense layer with the learned parameters **W**_out_ and **b**_out_. The token probabilities are adjusted by temperature *T*, where a lower *T* enforces deterministic sampling and a higher *T* increases randomness.

For molecule generation, recursion starts from

5 $$s_{0}=\mathrm{START},\;h_{0}=0,\;c_{0}=0$$

For fragment-based generation, predefined fragments smile_1_, ..., and smile_*N*_ sequentially update the LSTM states before proceeding with (1–4). Specifically, the initial states **h**_0_, **c**_0_ are iteratively updated using (1 and 2) for each fragment symbol smile_*i*_ before sampling new tokens. This ensures that the generated molecules incorporate the structural constraints imposed by the fragment.

### Molecular Validation

To evaluate the properties of the generated molecules, three key criteria were used: **validity, uniqueness, and novelty:**

- **Validity**: a generated molecule was considered valid if its molecular structure adhered to known chemical rules. The RDKit^35^ Python package was used to verify the correctness of the generated SMILES sequences.
- **Uniqueness**: a molecule was classified as unique if it was structurally distinct from all other valid molecules within the generated data set.
- **Novelty**: a molecule was considered novel if it did not appear in existing data sets, specifically the QM9 ^38^ data set and the RON regression model data sets, as determined by checking the presence of its generated SMILES string in these data sets.

Subsequently, developed fuel property regression models were used to predict density, boiling point, viscosity, and enthalpy of combustion for the most promising novel compounds, which were predicted to have a high RON. An acceptable range for each fuel physical property was suggested by considering gasoline specifications ASTM International,^41^ European Committee for 189 Standardization (CEN)^42^ but with extended limits so as not to preclude the inclusion of any generated novel molecules with potential as practical fuels when utilized as blending components or with additives. For example, an extended density range was considered so as not to preclude generated molecules containing multiple oxygen atoms. Therefore, only molecules that met the following physical property criteria were retained for further consideration: the enthalpy of combustion is equal to or greater than 25,000 kJ/kg, a boiling point of between 303 and 493 K, a density of less than 1000 mg/cm^3^, and a viscosity less than 1 mPa·S.

## Results

### Fuel Property Modeling

Figure 2 shows the best-performing regression models for fuel properties, such as RON, density, boiling point, viscosity, and combustion enthalpy. Models with larger data sets, such as viscosity (1554 data points), boiling point (5549 data points), and density (3,930 data points), generally had the lowest mean percentage error. For example, the boiling point predictions were highly accurate with a mean absolute percentage error (MAPE) of 0.01, an *R* ^2^ of 0.99, and a mean absolute error (MAE) of 2.64.

**Figure 2:**
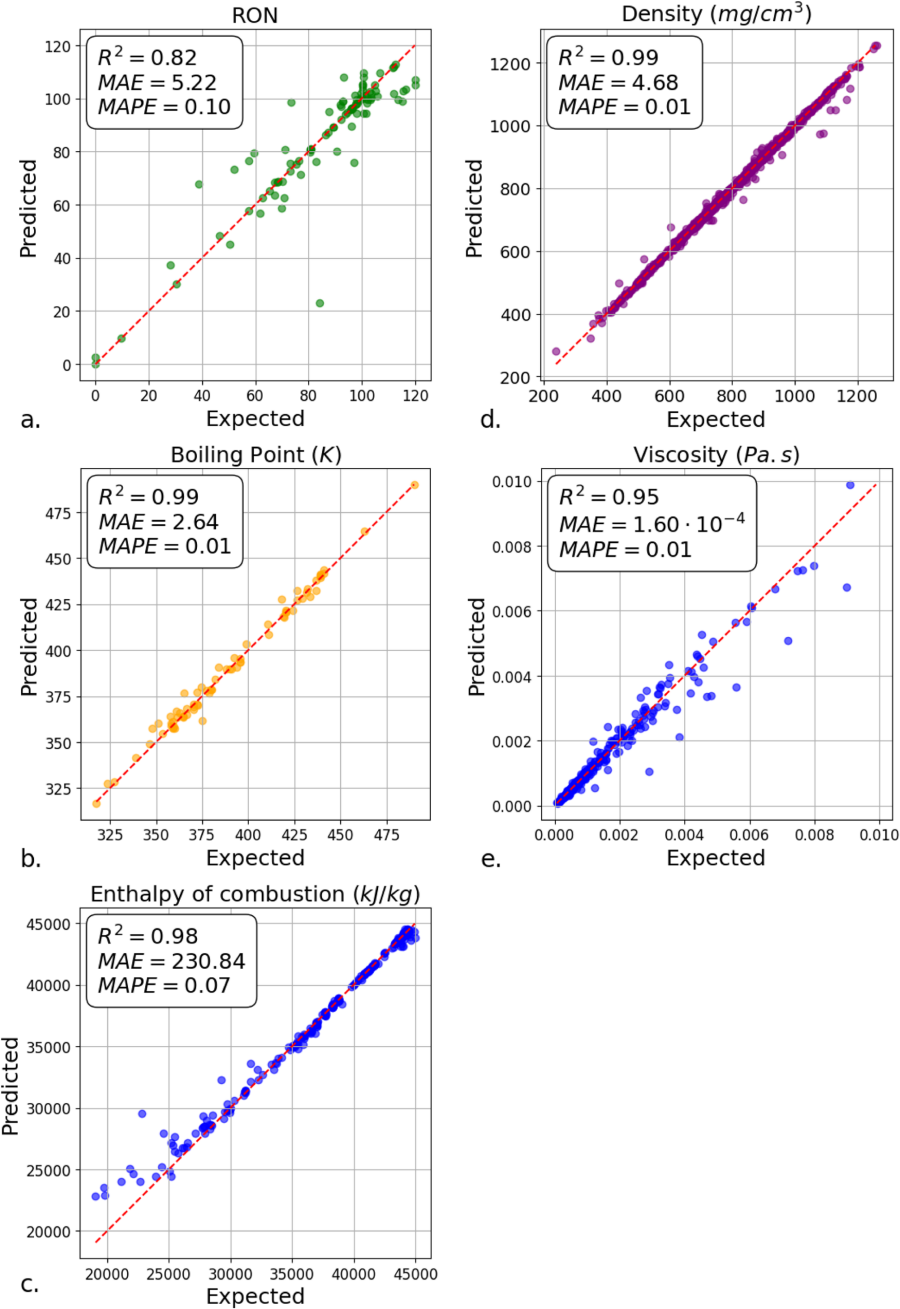
Fuel property modeling, showing predicted and expected values for (a) octane, (b) boiling point, (c) enthalpy of combustion, (d) density, and (e) viscosity.

Among all of the property prediction models developed, the RON model exhibited the weakest performance, which is attributed to its limited training set (362 unique data points). It showed higher prediction errors (MAE = 5.22, MAPE = 0.10) and lower explanatory power (*R* ^2^ = 0.82) compared to the other fuel property models. It can also be seen from Figure 2a that the model systematically underestimates the RON values for high-octane compounds, particularly ethyl butanoate, methyl acetate, and multisubstituted aromatics (*o*-xylene, *p*-xylene, and indene); further underpredicted molecules are identified in the Supporting Information, Figure S2. This bias can be attributed to the presence of these compounds in regions of chemical space far from most of the training set, leading to underprediction of two specific molecule groups: polar oxygenates and sterically hindered aromatics.

Substructure analysis of the underpredicted molecules revealed that this bias originates from critical deficiencies in the training set. For example, the underprediction of indene arises from the complete absence of fused aromatics in the training data; the low accuracy for methyl acetate and ethyl butanoate reflects the under-representation of esters (only 15 examples). Similarly, the systematic underestimation of *o*- and *p*-xylene is linked to the limited diversity of ortho- and meta-substituted aromatics (28 examples each), which hinders the model’s ability to learn substituent-position effects.

Although the model performs worse in terms of *R* ^2^ compared to vom Lehn et al.^43^ (*R* ^2^ = 0.91) and Schweidtmann et al.^44^ (*R* ^2^ = 0.94), it achieves satisfactory ranking ability (Spearman’s ρ = 0.89), which is sufficient for its primary role: guiding molecular optimization in the hill-climb algorithm. Future improvements should focus on:

- Expanding training data diversity, especially for oxygenates and polyfunctional aromatics
- Incorporating quantum-chemical descriptors to better capture electronic structure effects

The viscosity model showed a slight bias toward higher values by 0.004 Pa·s, attributed to a skewed data set that followed a logarithmic normal distribution. Specifically, 998 compounds had viscosities below 0.0002 Pa·s, compared to 556 with higher values. Furthermore, the viscosity was underpredicted for seven compounds, suggesting the presence of outliers.

Similarly, the enthalpy of the combustion model (2057 data points) exhibited bias for values between 20,000 and 25,000 kJ/kg, often overpredicting values. For instance, it predicted 24,000 kJ/kg when the expected value was 20,000 kJ/kg. The enthalpy data set included 61 compounds below 25,000 kJ/kg, with 1996 compounds unevenly distributed between 25,000 and 45,000 kJ/kg, yielding a mean of 39,941.08 kJ/kg and a standard deviation of 5923.42 kJ/kg.

However, density predictions showed the lowest variance among the models, indicating high reliability. In general, the characteristics of the data set, such as size, distribution, and potential outliers, significantly impacted the accuracy of the model and introduced biases. These findings underscore the importance of data quality and distribution in achieving reliable predictions of the fuel properties.

### Molecular Generation Analysis

Before the generative model was fine-tuned using Algorithm 1, it was validated by generating 1000 SMILES strings representing molecular structures at different sampling temperatures (eq 3). Figure 3 illustrates the ratio of generated molecules that are valid and novel. The generative model achieved a maximum validity of 0.971 at a temperature of 0.8 and a maximum novelty of 0.456 at a temperature of 1.8.

**Figure 3:**
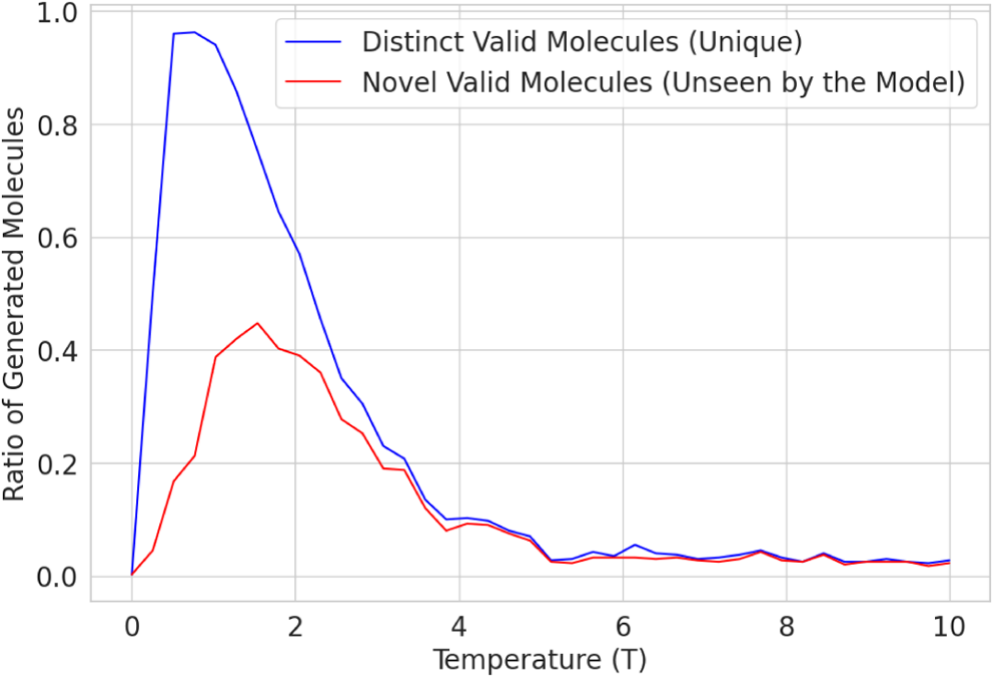
Unique and novel molecules vs sampling temperature *T*.

These results highlight the influence of the sampling temperature on the diversity of generated molecular structures. At a lower temperature of 0.8, the model produces many valid molecules, 0.971, suggesting that it primarily generates well-learned patterns from the training data. In contrast, at a higher temperature of 1.8, the model explores less frequent patterns, increasing novelty by 0.456 but likely reducing validity. This trade-off emphasizes the importance of temperature tuning in balancing the molecular diversity and reliability.

Figure 4 presents the distribution of modified RON in hill-climbing iterations (Algorithm 1), with step zero representing the initial state of the model. Negative RON prediction values were assigned to valid compounds containing atoms uncommon in hydrocarbons or oxygenated fuels such as nitrogen and fluorine. The algorithm converged at step 9, generating compounds with a mean RON value of 78.89 and a median value of approximately 95. Despite convergence, the mean RON value remained relatively low, likely because of the continued generation of outlier compounds containing nonfuel atoms.

**Figure 4:**
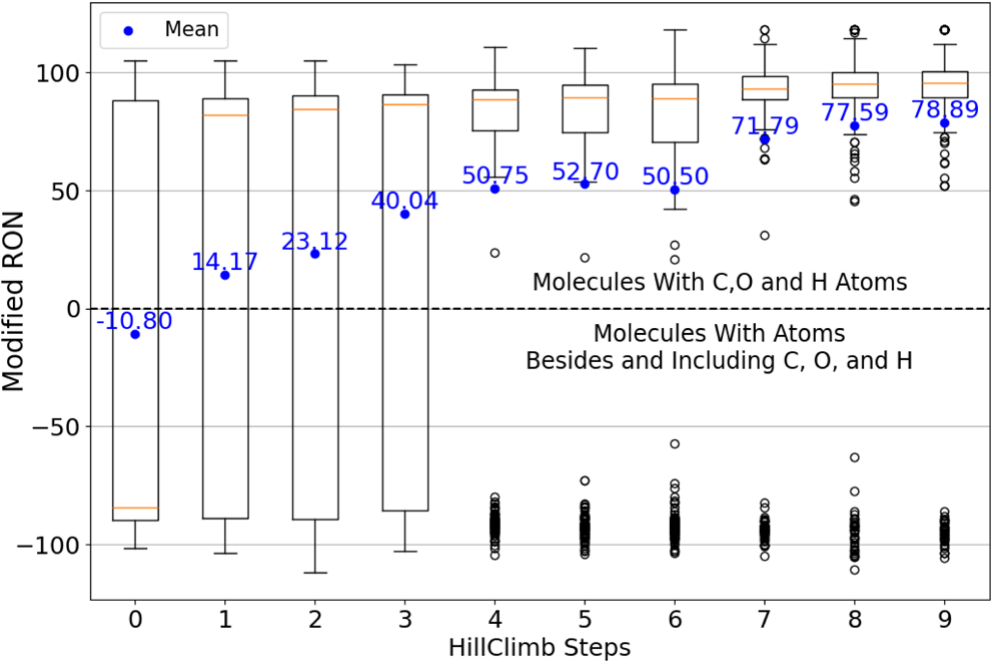
Fine-tuning of the generative model via hill climbing.

Subsequently, the model generated 500 compounds, ranked by predicted RON using the same model used in the hill-climbing reward function. Among the 30 compounds with the highest ranking, only five were novel and passed the filtering of physical properties, meaning they were absent from both the QM9 ^38^ training data set and the RON data set used for fine-tuning (Algorithm 1). Figure 5 illustrates the composition of the generated compounds at each stage of the filtering process.

**Figure 5:**
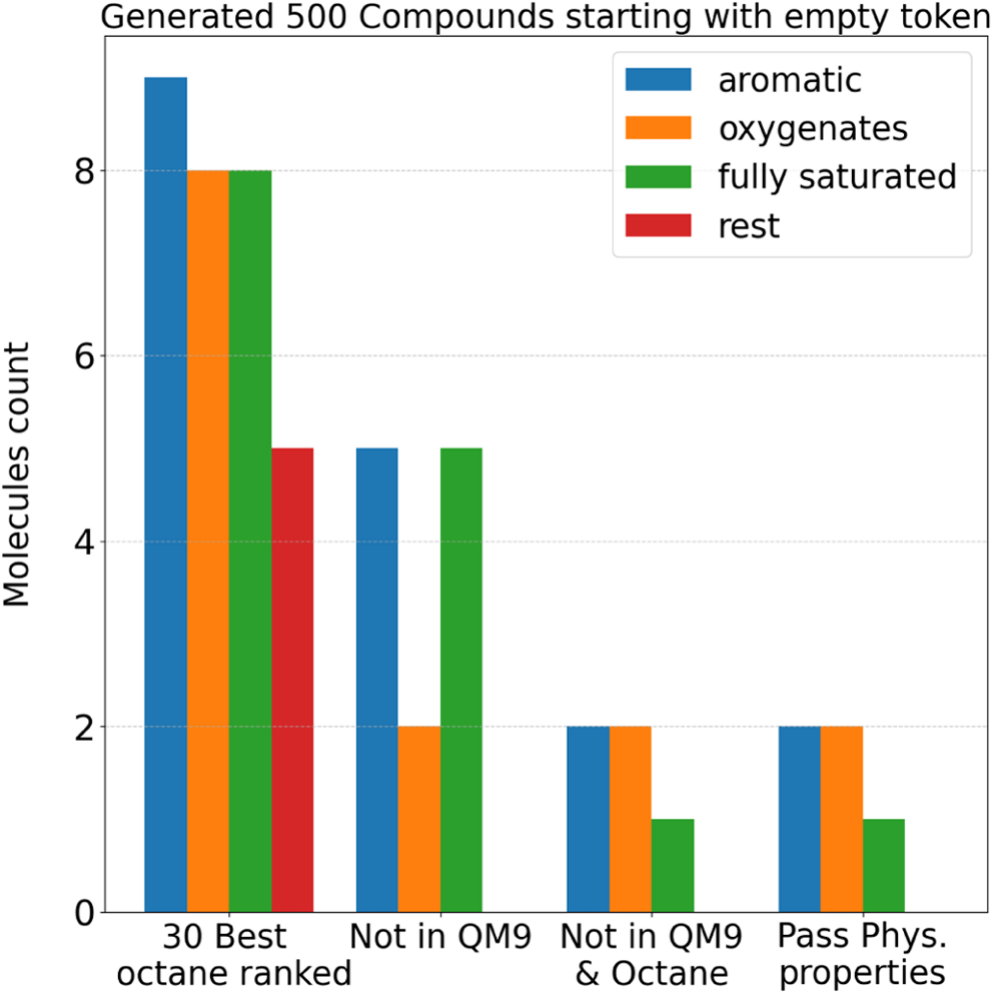
Composition of the molecular structures at each filtering step.

The 30 best-performing molecules are included in the Supporting Information (Figure S1). An unusual feature in these molecules was the presence of a cyclopropyl-containing structure, such as 1-tert-Butyl-1-methylcyclopropane (Figure S1. 21), incorporating triangular motifs that are less common in practical fuels (and which were present in only a limited number of the training data set compounds). Furthermore, while methanol, a widely considered alternative fuel for spark ignition engines,^45^ was identified, other methyl-oxygenates of interest, for example, dimethyl carbonate and methyl formate,^46,47^ were not present in the 30 best-performing molecules (Figure S1).

### Molecular Structures

Figure 6 presents the five novel molecules and their predicted physical properties. The generated molecules exhibit a high degree of compactness and branching. For instance, 2,3,3,4-tetramethyl shares structural similarities with 2,2,3,3-tetramethylpentane, a compound with a known research octane number of 114. Furthermore, two of the generated molecules contain aromatic rings, which are known to enhance knock resistance.^48,49^ The smallest identified molecule, 3-methylbutan-2-ol, includes an alcohol functional group, which can influence combustion characteristics.

**Figure 6:**
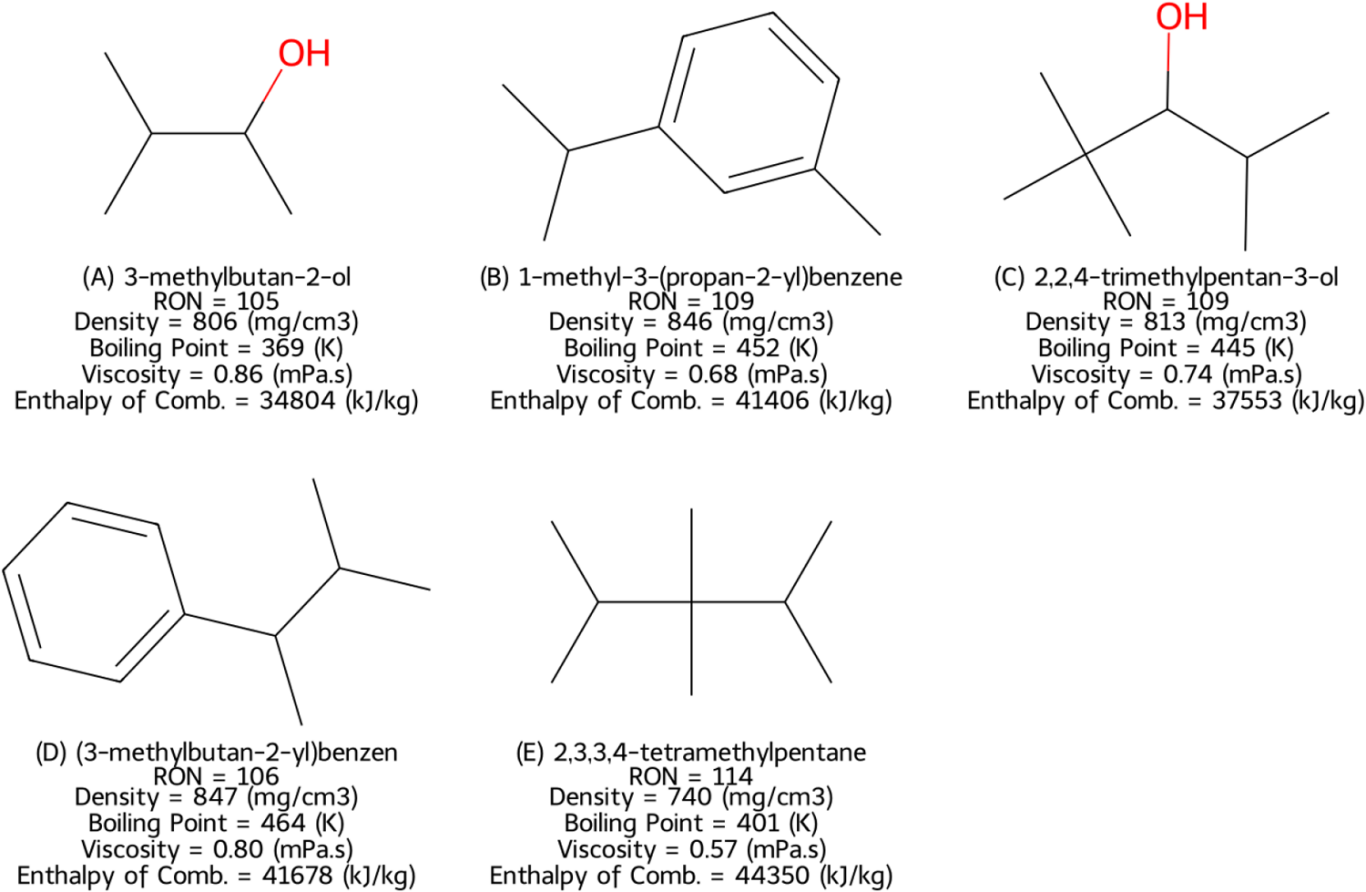
Generated molecules (empty token), SMILES absent in QM9 ^38^ and RON data sets.

To further explore oxygen incorporation in biofuel-like structures, the trained model generated 3000 compounds, each initialized with an oxygen atom to ensure its presence in the final molecular structures. Increasing the number of generated molecules from the original 500 to 3000 aimed to improve structural diversity while adhering to the oxygen constraint. Figure 7 showcases the oxygen-containing molecules that were absent from both the QM9 ^38^ training data set^38^ and the RON fine-tuning data set.

**Figure 7:**
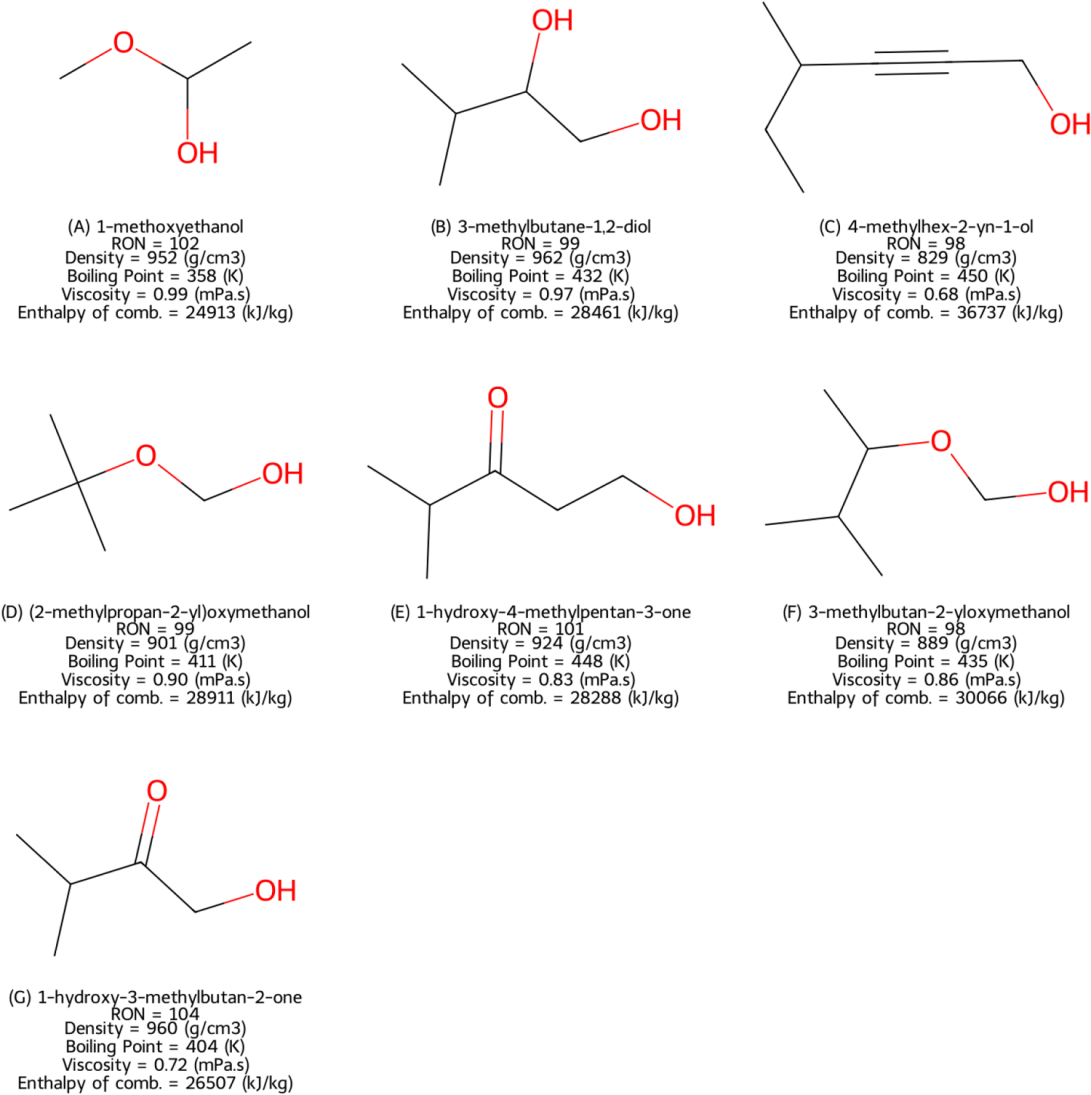
Generated molecules (oxygen token), SMILES absent in QM9 ^38^ and RON data sets.

Compared with the unconstrained generation in Figure 6, a notable reduction in aromatic species was observed. Instead, most of the generated compounds contained multiple oxygen atoms and exhibited increased branching. The molecules also displayed a broader range of oxygen-bearing functional groups, including ketones and ethers, in Figure 7. The presence of ether linkages is significant, as they are known to influence RON.

Meanwhile, the presence of the hydroxyl group can be seen to increase the fuel viscosity. For example, 1-methoxyethanol (Figure 7A) and 3-methylbutane-1,2-diol (Figure 7B) exhibited borderline viscosity values, which were chosen as the threshold for physical property checks. Furthermore, some structures, such as 4-methylhex-2-yn-1-ol (Figure 7C), feature a triple bond, which can affect both RON and soot formation.

## Discussion

### Properties of Generated Fuel-like Compounds

Two of the five remaining molecules generated from the empty token (eq 5), shown in Figure 6B,D, were aromatic. These molecules were also highly branched, a combination of structural properties known to increase RON.^50^ A similar trend can be observed in molecules such as 3-methylbutan-2-ol (Figure 6A) and another example in 2,2,4-trimethylpentan-3-ol (Figure 6C), both of which feature two structural properties associated with higher octane numbers: the presence of an alcohol group and a highly branched structure. The remaining molecule, depicted in Figure 6, also exhibited highly branched characteristics. In addition, the compound predicted that RON values were not beyond the typical range for the hydrocarbons. In general, the compounds of Figure 6 did not have unfamiliar molecular structural features.

The predicted combustion enthalpy was the lowest for 3-methylbutan-2-ol (Figure 6A) and 2,2,4-trimethylpentan-3-ol (Figure 6C), with values of 34,804 kJ/kg and 37,533 kJ/kg, respectively. This result is consistent with the expectation that the calorific value of the molecules decreases with an increase in the number of oxygen atoms.

The other set of generated molecules, starting with the oxygen symbol in Figure 7, exhibited considerably lower predicted combustion enthalpies compared to the molecules in Figure 6, again due to the abundance of oxygen atoms. As expected, the molecule in Figure 7C (4-methylhex-2-yn-1-ol) had the highest combustion enthalpy; it contains only one oxygen atom.

From the RON perspective, the molecules in Figure 7 exhibited high branching and the presence of an alcohol group, both of which are known to contribute to higher octane numbers. However, the molecules (2-methylpropane-2-yl)­oxymethanol in Figure 7D and 3-methylbutan-2-yloxymethanol in Figure 7F included ether linkages, which are known to decrease RON. This result was unexpected, given the objective of the generative model.

Interestingly, once the ether linkage is broken in these molecules, the resulting fragments include ethanol (RON 108) and 2-methylpropane (RON 92) for the molecule in Figure 7D and ethanol (RON 108) and 2-methylbutane (RON 93) for the molecule in Figure 7F.

### Practical Applications and Implications for Fuel Property Optimization

The aromatics in Figure 6B,D can be synthesized through various methods, including pyrolysis, for the processing of lignocellulosic biomass.^51^ The other molecules in Figure 6 require additional postprocessing steps, including hydrogenation at high temperatures.^52^ Meanwhile, the branched alcohol shown in Figure 6A is an isomer of isoamyl alcohol, which is industrially produced by microbial fermentation.^53^

Regarding oxygenates (Figure 7), short-chain oxygenates can also be produced from biomass via pyrolysis. The molecule 3-methylbutane-1,2-diol (Figure 7B) can be produced from cellulose.^54^ The molecules 1-hydroxy-3-methylbutan-2-one (Figure 7G) and 1-hydroxy-4-methylpentan-3-one (Figure 7E) are fatty acids, which are naturally produced by plants.^55^ 4-Methylhex-2-yn-1-ol (Figure 7C) is difficult to produce, as it requires the removal of hydrogen from a triple bond, which is a complex process. Furthermore, the presence of the triple bond may lead to soot formation, as was shown by Ladommatos et al.^56^ The short-chain ethers (Figure 7D,F) can, however, be produced via etherification of various compounds from renewable feedstocks, for example, alcohols and carbon dioxide.^57^

### Limitations and Future Directions

This section outlines the limitations of this study and proposes future directions. The primary limitations arise from the data set, the generative modeling approach, and the evaluation of the generated compounds. A significant limitation is the RON data set, which provides reliable predictions primarily for a narrow range of compounds and lacks a wide variety of oxygenates, which are the primary types of biofuels. Consequently, the performance of the fuel design algorithm is influenced by the predictive accuracy of the RON model, particularly for high-octane oxygenates. Nonetheless, the proposed framework is model-agnostic and can be readily adapted to more accurate RON predictors as they become available, thereby mitigating this limitation in future applications.

Currently, molecules are generated by using a left-to-right SMILES representation. This approach may limit the diversity of the compounds generated, especially when the generation process starts with a predefined molecular fragment. To address this, adopting bidirectional LSTMs,^58^ which process sequences in both directions (left-to-right and right-to-left), could enhance compound diversity. However, an advantage of the current approach utilizing SMILES strings for the representation of the generated molecules is that language models are inherently quick to perform text generation, reducing the computational intensity of the process.

Additionally, the hill-climbing algorithm used to fine-tune the generative model ranks compounds solely on the basis of research octane number predictions. As a result, structural optimization does not take physical properties. Therefore, the ranking process should consider physical properties by first filtering compounds on the basis of these properties and then ranking them according to their octane predictions.

Another limitation is that the current physical property filtering does not include key properties, such as miscibility, which are essential for evaluating the real-world applicability of the generated molecules. Incorporating these properties into the filtering process would ensure that the generated compounds meet both the desired octane number and other critical physical constraints.

This study is based on the use of single-component hydrocarbons, some containing oxygen, and it is acknowledged that when single components are blended, the resulting knock resistance is not an average of the individual RON values due to nonlinearities and synergies between the fuel molecules.

Finally, some generated compounds lack clear synthesis pathways. This issue could be addressed by starting molecular generation from predefined fragments that are already recognized as intermediates in material processing, particularly those with potential as fuel precursors.

## Conclusions

This research established a baseline for the generation of fuel compounds using a simple yet effective autoregressive model combined with the hill-climb algorithm, which has been shown to outperform graph-based generation in some cases by Brown et al.^14^ In contrast to other studies, this approach considered the physical properties of the generated compounds and excluded molecules that were either already present in the training data or found in the octane data sets. The results showed that only 5 of the top 30 highest-ranked compounds (from a total of 500 generated molecules) had reasonable fuel-like physical properties, such as density, viscosity, boiling point, and enthalpy of combustion, and were not present in the training data or the RON regression model data set. Furthermore, for molecules generated using the oxygen token, 7 of the top 30 compounds ranked on the basis of the RON predictions displayed reasonable physical properties and were not present in the octane and QM9 ^38^ data sets. However, the remaining compounds after filtering displayed structural features expected from high-knock-resistant compounds.

## Supplementary Material





## Notes

Complete RON data set (362 compounds) containing SMILES strings, experimental values, references, and training split labels (XLSX)
Hyperparameters of final fuel property prediction models (SVM, GBM, MLP) (Table S1); top 30 generated molecules ranked by predicted RON, with experimental/QM9 annotations (Figure S1); and high RON outliers underpredicted by the model (Figure S2) (PDF)
