# Supplementary material for: Generation of Novel Fuels Optimized for High-Knock Resistance with a Long Short-Term Memory Model
Source: Energy Fuels. 2025 Jun 30;39(27):13044–53. doi: 10.1021/acs.energyfuels.5c01155 (PMC12257456; doi:10.1021/acs.energyfuels.5c01155)
Supplement: Supplementary file 2 [file ef5c01155_si_002.pdf]

Supporting Information: Generation of Novel Fuels Optimised for High-Knock Resistance with a Long Short-Term Memory Model

Sergey Anufriev,* Paul Hellier, and Nicos Ladommatos

Department of Mechanical Engineering, University College London, Roberts Building, Torrington Place, London, WC1E 7JE, UK

E-mail: sergii.anufriiev.10@alumni.ucl.ac.uk;anufriiev.sergii@gmail.com

Supporting Information Available

The following Supporting Information is available:

- **Table S1:** Final fuel property prediction models and their hyperparameters, selected using the Optuna optimizer from the parameter search space described in Table 2 of the main manuscript.
- **Figure S1:** The top 30 generated molecules ranked by predicted RON values, with indications of experimental RON values (where available) and inclusion in the QM9 dataset.
- **Figure S2:** Outliers in high-RON predictions, particularly those with experimental RON values above 110 that were underpredicted by the model.
- **Excel file:** Full RON dataset of 362 compounds used in this study.

Supplementary Tables

Table S1: Final selected models and their hyperparameters. All models were implemented using scikit-learn.

Fuel Property	Model	Hyperparameters
Octane Number	SVM	$\texttt{C} = 664$, $\texttt{degree} = 10$, $\texttt{gamma} = 0.099$
Enthalpy of Combustion	SVM	$\texttt{C} = 999$, $\texttt{degree} = 3$, $\texttt{gamma} = 0.056$
Density	GBM	$\texttt{max\_depth} = 6$, $\texttt{max\_features} = 27$, $\texttt{n\_estimators} = 249$
Boiling Point	MLP	$\texttt{alpha} = 1.341 \times 10^{-4}$, $\texttt{batch\_size} = 7$, $\texttt{learning\_rate} = 0.000978$
Viscosity	SVM	$\texttt{C} = 993$, $\texttt{degree} = 4$, $\texttt{gamma} = 9.9456 \times 10^{-3}$

Supplementary Figures

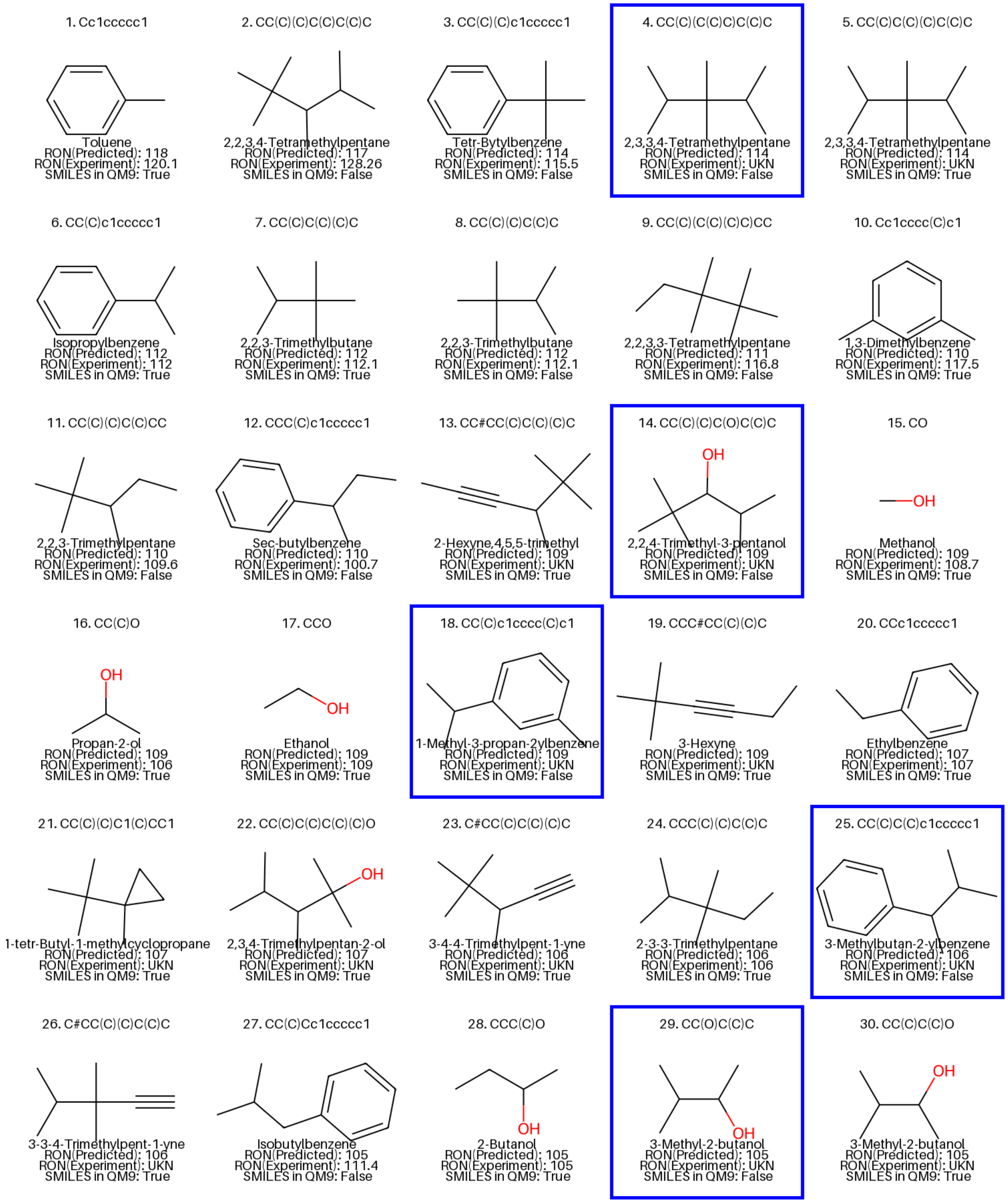

Figure S1: Top 30 molecules ranked by predicted Research Octane Number (RON). Experimental RON values are indicated for molecules with available data from the RON dataset.xlsx; molecules without such data are labeled as "UKN." Inclusion of each molecule in the QM9 dataset is also indicated. Highlighted structures correspond to those selected for Figure 6: generated molecules with empty tokens and SMILES absent from both the QM9 and RON datasets

	RON	Predicted_RON	Absolute Error	Compound Name	SMILES	Molecule
**0**	115.4	99.412573	15.987427	Ethyl buanoate	CCCC(=O)OCC	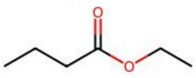
**1**	120.0	104.121308	15.878692	Methyl Acetate	CC(=O)OC	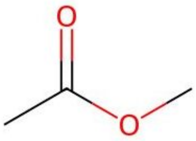
**2**	120.0	105.044321	14.955679	O-Xylene	CC1=CC=CC=C1C	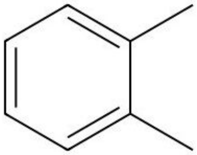
**3**	113.7	99.211079	14.488921	indene	c1ccc2c(c1)C=CC2	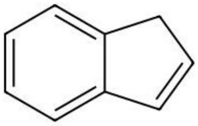
**4**	116.4	103.500415	12.899585	P-Xylene	Cc1ccc(cc1)C	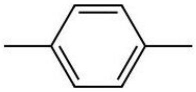
**5**	115.5	102.900056	12.599944	m-diethylbenzene	CCc1cccc(c1)CC	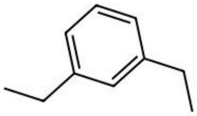
**6**	112.1	101.900163	10.199837	1-methyl-3-n-propylbenzene	CCCc1cccc(c1)C	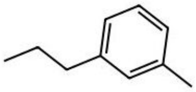

Figure S2: Outliers in the high-RON region. These molecules have experimental RON values greater than 110 but were underpredicted by the model. They correspond to compounds visible in Figure 2.a of the main manuscript (expected vs. predicted RON). Potential causes include limited training examples in this range.

RON Values Dataset

The complete data set of 362 molecules used in this study is provided in the accompanying Excel file (`RON_dataset.xlsx`). Each entry includes the compound name, SMILES string, corresponding experimental RON value, source reference, and a column indicating whether the molecule was included in the training split.
